# Deep RNA-seq of male and female murine sensory neuron subtypes after nerve injury

**DOI:** 10.1097/j.pain.0000000000002934

**Published:** 2023-06-06

**Authors:** Allison M. Barry, Na Zhao, Xun Yang, David L. Bennett, Georgios Baskozos

**Affiliations:** Nuffield Department of Clinical Neurosciences, University of Oxford, Oxford, United Kingdom

**Keywords:** Neuropathic pain, Sexual dimorphism, RNA-seq, DRG, DRG subtypes, Transcriptomics resource

## Abstract

Dorsal root ganglia (DRG) neurons have been well described for their role in driving both acute and chronic pain. Although nerve injury is known to cause transcriptional dysregulation, how this differs across neuronal subtypes and the impact of sex is unclear. Here, we study the deep transcriptional profiles of multiple murine DRG populations in early and late pain states while considering sex. We have exploited currently available transgenics to label numerous subpopulations for fluorescent-activated cell sorting and subsequent transcriptomic analysis. Using bulk tissue samples, we are able to circumvent the issues of low transcript coverage and drop-outs seen with single-cell data sets. This increases our power to detect novel and even subtle changes in gene expression within neuronal subtypes and discuss sexual dimorphism at the neuronal subtype level. We have curated this resource into an accessible database for other researchers (https://livedataoxford.shinyapps.io/drg-directory/). We see both stereotyped and unique subtype signatures in injured states after nerve injury at both an early and late timepoint. Although all populations contribute to a general injury signature, subtype enrichment changes can also be seen. Within populations, there is not a strong intersection of sex and injury, but previously unknown sex differences in naïve states—particularly in Aβ-RA + Aδ-low threshold mechanoreceptors—still contribute to differences in injured neurons.

## Abstract 2

Supplemental Digital Content is Available in the Text.

Using sex, injury, and time factors, RNA-seq was performed on dorsal root ganglia subtypes isolated through fluorescent activated cell sorting. This was analyzed and curated as a resource for others.

## 1. Introduction

Chronic pain conditions affect around 25% of the global population. Neuropathic pain, as a subclass, affects ∼8%. It is directly tied to nervous system damage through trauma, disease, or therapeutic use (eg, chemotherapy and antiretrovirals).^15,16^ Current treatment options are widely considered inadequate, and quality of life scores remain significantly reduced.^4,33,53^ Increasing lifespans, diabetic prevalence, and decreases in cancer mortality are all contributing to increases in these disorders, adding weight and urgency to deepen our understanding.^15,33^

Primary afferent pathophysiology is believed to be a key driver for peripheral neuropathic pain disorders, with dorsal root ganglia (DRG) neurons being well described for their role in driving both acute and chronic pain. These neurons encompass a diverse collection of subtypes that are grouped by various related factors, including size, myelination, conduction velocity, projection patterns, end organ innervation, and functional properties.^23,30^

More recently, single-cell or nuclear RNA-seq in mice^35,51,78,86^ and humans^50,72^ have emphasized the diversity within DRG ganglia. Gene expression differs between these broad subpopulations and is indeed predictive of their functional properties.^88^ This diversity is lost during bulk RNA-seq, due to the consolidation of subtypes together. In single-cell data sets, pseudo-bulk samples can be generated, but this relies on a well-defined clustering that can be lost after nerve injury.^32,51^ As such, changes at a subtype level remain unclear in painful states and are the focus of the current study.

Understanding sexual dimorphism is also a fundamental clinical issue. Females are more likely to be living with chronic pain, and treatment efficacy can be sex dependant.^27,46,47^ In naïve states, quantitative sensory testing has also highlighted heightened pain sensitivity in females.^9^ The case for studying sexual dimorphism is not new,^11,77^ but historical biases within the research community resulted in a predominantly male focus.^47,65,80^

More recent female-inclusive studies have revealed clear mechanistic differences between sexes. Sorge et al.^68,69^ report a prominent sex difference when studying mechanical hypersensitivity: While males depend on the microglia activity, female animals depend on adaptive immune cells. Brain-derived neurotrophic factor (BDNF) and prolactin both affect pain in a sex-dependant manner,^48,57^ and there is evidence of higher level dimorphisms affecting pain percepts.^43^ At a transcript level, sexual dimorphism is also visible,^10,44^ with differences seen in human DRG as well.^52,72^

Here, we build on this through the deep transcriptional profiles of murine DRG populations in acute and late pain states while considering sex differences. We have studied 5 populations: *Scn10a*-expressing, peptidergic (PEP) and nonpeptidergic (NP) nociceptors, as well as C-low threshold mechanoreceptors (C-LTMRs) and *Ntrk2*-expressing A-LTMRs. In naïve states, we find subtype-specific sexual dimorphism in a small number of genes. This does not translate to a strong interaction of sex and injury at the neuronal transcript level. We also see both stereotyped and unique signatures in injured states, with notable changes in C-LTMR and NP populations, as well as a distinct transcriptional program in Aβ-RA + Aδ-LTMRs compared with the other subtypes in the study. A searchable database is available at https://livedataoxford.shinyapps.io/drg-directory/.

## 2. Methods and materials

All work was conducted in accordance with the UK Home Office and the University of Oxford Policy on the Use of Animals in Scientific Research. This study conforms to ARRIVE guidelines.

Animals were housed in standard conditions on a 12-hour light/dark cycle with food and water ad libitum. All animals were randomly assigned to experimental groups where applicable. Internal controls were used when not possible to randomize (ie, ipsilateral vs contralateral comparisons). Unless explicitly stated, all experiments were performed on both males and females. In brief, driver lines were bred with a fluorescence reporter for various experiments. When necessary, inducible lines were dosed with intraperitoneal (i.p.) injection(s) of tamoxifen. Specific details are mentioned below.

### 2.1. Transgenic details

C57BL/6 mice were purchased from the Oxford University Breeding Unit. Cre driver lines used include the following: Calca^tm1.1(cre/ERT2)Ptch^ (CGRP, gifted from Prof. Pao-Tien Chaung),^67^ Mrgprd^tm1.1(cre/ERT2)Wql^/J (MRGPRD, JAX 031286),^54^ Scn10a^tm2(cre)Jnw^ (Nav1.8, gifted from Prof. John Wood),^49^ Th^tm1.1(cre/ERT2)Ddg^/J (TH, gifted from Prof. David Ginty),^81^ and Ntrk2^tm1.1(cre/ERT2)Ddg^/J (TRKB, gifted from Prof. Paul Heppenstall).^20^ Cre-driver lines were bred and maintained as heterozygotes, except for Th^creERT2^, which was bred as homozygous. Details are listed in Table 1.

**Table 1:** Transgenic lines used in the current study.

Mouse	Source	Identifier
Calca^tm1.1(cre/ERT2)Ptch^	gift: Pao-Tien Chuang	67
Mrgprd^tm1.1(cre/ERT2)Wql^/J	The Jackson Laboratory	54, CAT#:031286
Scn10a^tm2(cre)Jnw^	gift: John Wood	49
Tg(Ntrk2-cre/ERT2)\#Phep	gift: Paul Heppenstall	20
Th^tm1.1(cre/ERT2)Ddg^/J	gift: David Ginty	81, CAT#:025614
Advillin^flpO^	gift: David Ginty	81
B6.129S-Gt(ROSA)26Sor^tm32(CAG-COP4*H134R/EYFP)Hze^/J	gift: Simon Butt	40, CAT#:012569
B6.Cg-Gt(ROSA)26Sor^tm14(CAG-tdTomato)Hze^/J	The Jackson Laboratory	41, CAT#:007914
B6.Cg-Gt(ROSA)26Sor^tm80.1(CAG-COP4*L132C/EYFP)Hze^/J	The Jackson Laboratory	18, CAT#:025109

The following reporters were used to visualize DRG subpopulations: B6.129S-Gt(ROSA)26Sor^tm32(CAG-COP4*H134R/EYFP)Hze^/J (JAX 012569, gifted from Prof. Simon Butt), tdTomato B6.Cg-Gt(ROSA)26Sor^tm14(CAG-tdTomato)Hze^/J (JAX 007914), and ai80D B6.Cg-Gt(ROSA)26Sor^tm80.1(CAG-COP4*L132C/EYFP)Hze^/J, (JAX 025109). Ai32 and ai14 use was based on initial breeding availability. Ai80 depends on both Flp and Cre recombinase for intersectional targeting and was used for neuronal targeting of *Ntrk2*. Ai80 was first crossed to an Advillin^flpO^ (Transgenic line, bred for experiments as needed, gifted from Prof. David Ginty). Reporters were bred as homozygotes where applicable. Advillin^flpO^ was bred to a C57BL/6 background for at least 7 generations before experimental use.

### 2.2. Tamoxifen regimes

Tamoxifen (Sigma-Aldrich) was dissolved 20 mg/mL in corn oil through sonification. All animals were dosed i.p., and health statuses were monitored daily for the duration of the dosing regime. Calca^creERT2^ was dosed 5x (daily) with 75 mg/kg in adulthood. Mrgprd^creERT2^ was dosed 5x i.p. (0.5 mg/animal/day), beginning between P10-P17. Body weight recovered more quickly when dosed at later stages, with no noticeable difference in reporter expression. We recommend dosing begin at P17 for this line moving forward. Th^creERT2^ was dosed 1x with 50 mg/kg above 6 weeks of age. Ntrk2^creERT2^ was dosed 5x (daily) with 75 mg/kg in adulthood.

### 2.3. Spared nerve injury

Adult mice were anesthetized with 2% inhaled isoflurane. Using the sterile technique (including incision site sterilization and surgical drapes), the sciatic nerve was exposed before ligation and transection of the tibial and common peroneal branches.^19^ The sural nerve was left intact. Each animal was dosed with systemic (5 mg/kg Rimadyl, Pfizer) and local (2 mg/kg Marcaine, AstraZeneca) postoperative analgesia. Animals were monitored daily for self-mutilation, and no animals required sacrifice due to tissue damage.

### 2.4. Sample collection

The sample size was calculated using the algorithm published by Zhao et al.^87^ See supplemental methods for full details (available at http://links.lww.com/PAIN/B823). In total, 160 paired samples were collected (ipsilateral and contralateral) over 5 neuronal subtypes at an acute (3 day) and late (4 week) timepoint after spared nerve injury (SNI). Ipsilateral lumbar (L3-L5) were compared with contralateral (L3-L5) DRGs to ensure an internal control. One animal was used for each sample pair, excluding Th^creERT2^, where DRGs from 2 animals were pooled during dissection. Male and female samples were evenly split, with the exclusion of Mrgprd^creERT2^, 3 days after SNI where only 3 female mice could be used. Five males were thus processed for this group.

Multiple animals were processed in parallel, but collection times from perfusion to frozen were kept to less than 4 hours. Adult animals were first overdosed with pentobarbital and perfused transcardially with sterile, ice cold saline. Lumbar DRG were quickly removed and placed into Hanks' Balanced Salt Solution (HBSS) on ice. Postdissection of all tissue, collagenase/dispase was added for a 60 minutes digest at 37°C followed by mechanical dissociation with polished glass pipettes. Myelin and debris were reduced using a clean 15% w/v bovine serum albumin (BSA) cushion. Samples were placed on ice and centrifuged at 4°C as much as possible (ie, excluding digestion). Before Fluorescence-Activated Cell Sorting (FACS), a subset of neurons from each sample was examined under bright field.

### 2.5. Library preparation and sequencing

Samples were transferred on ice immediately to the WIMM FACS Facility (Oxford) for sorting on a BD FACSAria Fusion 1 or Fusion 2 (Supplemental Figure 1, available at http://links.lww.com/PAIN/B823). For each condition, 100 cells were isolated through a single cycle, directly into low protein-binding Eppendorfs containing 2 ul NEBNext Single Cell Lysis Buffer (NEB, E5530S). Samples were kept on dry ice until transfer to −80°C for overnight storage.

Once all samples were collected, samples were thawed on ice, vortexed, and randomized into a 384-well 4titude FrameStar skirted PCR plate (Brooks Life Science, 4ti-0384/C; Thermo Scientific, AB-0558). Nondirectional libraries were prepared together using NEB Ultra low/Smarter library prep, as per manufacture instructions by the Oxford Genomics Centre at the Wellcome Trust Centre for Human Genetics. Libraries were amplified (21 cycles) on a Tetrad (Bio-Rad) using in-house unique dual indexing primers (based on^34^). Individual libraries were normalized using Qubit, and the size profile was analysed on the 2200 or 4200 TapeStation before pooling together accordingly. The pooled library was diluted to 10 nM for storage. The 10 nM library was denatured and further diluted before loading on the sequencer. Sequencing was performed over 3 independent runs and merged after quality control. Paired end sequencing was performed using a NovaSeq6000 platform using the S2/S4 reagent kit v1.5. Samples were sequenced with a 150 bp read length, at a depth of 30 million reads per sample. Raw data are available on Gene Expression Omnibus (GEO) (GSE216444).

### 2.6. Analysis

#### 2.6.1. Overview

Reads were mapped to the GRCm38 (mm10) Mouse Genome using STAR alignment.^21^ Samtools was used to sort, index, and merge Binary Alignment Map (BAM) files.^36^ Quality control (QC) was performed with both FastQC and Samtools before gene counting with HTSeq.^2,3,36^ Software is listed in Table 2.

**Table 2:** Software used in the current study.

Resource	Source
FastQC	3
Samtools	36
HTSeq	2
STAR	21
DESeq2 v1.32.0	38
Seurat – 3.2.0	13
R v4.1.0	https://www.r-project.org/
Cellranger	10X Genomics
ggplot2	83
complex heatmaps	28
clusterProfiler	84
msigdbr	22
Limma	61
pathfindR	76
goSeq	85
MultiQC v1.9	24
GraphPad Prism	www.graphpad.com

#### 2.6.2. Quality control

Samples were judged based on library size, as well as read assignment, alignment, and normalized gene coverage. Together, 6 samples were removed from downstream analyses, with details in the supplemental.

#### 2.6.3. DESeq2

Counts were corrected for effective library size in R using DESeq2.^38^ Normalized gene counts were fitted to a negative binomial distribution. A batch effect was introduced during sample collection, and a model that included this batch effect was fitted to every gene. The significance of the model's coefficients was assessed using the Wald test.

Counts were log transformed through variance stabilizing transformation (VST). Variance stabilizing transformation transformed counts were used for all plotting, unless otherwise stated. The R package Limma was used to remove the batch effect in principal component analysis (PCA) and heat map figures. Uncorrected PCA plots are shown in Supplemental Figure 2A-B, available at http://links.lww.com/PAIN/B823. Box plots show median + interquartile range (IQR), with 1.5 × IQR whiskers. Principal component analysis was performed using the top 5000 ENSEMBL genes ranked by standard deviation. Sample distances are proportional to the Mahalanobis distance, and ellipses show the 95% confidence interval of a condition's gene expression distribution. Hierarchical clustering was performed on transformed counts using Euclidean distances and complete linkage. Gene enrichment within neuronal subtypes was calculated using VST counts and was defined as genes with a subpopulation mean within the top 75% of expressed genes, across contralateral samples.

#### 2.6.4. Custom Gene Set Enrichment Analysis

Deep RNA-seq of naïve DRG subpopulations has been previously performed elsewhere.^88^ These results were curated into subpopulation-enriched gene sets to probe enrichment in the current data, with full details in the supplementals. In brief, RNA-seq count data were accessed from https://www.ncbi.nlm.nih.gov/geo/ (GSE131230). Expression data were generated by STAR alignment and HTSeq on the same genome build. Counts were corrected for library size and transformed by rlog in R using DESeq2 and filtered to match their published report. Within each subpopulation, genes with a mean rlog above the 95% quantile cut-off were curated into a “gene set” for subpopulation enrichment.

A second collection of gene sets was built from a recent study on the proteomic and transcriptomic enrichment of *Scn10a* neurons using a DTA-ablation model to compare signatures.^64^ Here, gene sets were based on significantly enriched or depleted candidates (log_2_ fold changes (LFC) > 1, false discovery rate (FDR) < 0.05) compared with control mice (males). To increase the power of the proteomic enrichment comparison by increasing the size of the gene set, more relaxed criteria were also examined (LFC > 0.5, FDR < 0.05).

These custom gene sets were then compiled for a Gene Set Enrichment Analysis (GSEA) analysis to our contralateral (“naïve”-like) samples using the clusterProfiler package in R using ranked fold changes from mean(subpopulation)/mean(total).

#### 2.6.5. Differential expression testing

Differential expression testing was performed on filtered data using the Wald test and a weighted FDR correction (independent hypothesis weighting (IHW)). Effect sizes were calculated using Bayesian shrinkage estimators (the *apeglm* method, through DESeq2) and are presented as moderated (shrunken) LFC,^89^ with full details in the supplementals. Significance was set at an FDR < 0.05 and an LFC > 1.

GSEA analyses against “all gene sets” were performed using ranked LFC through msigdbr^22^ and clusterProfiler^84^ libraries. Custom GSEA analyses were calculated against a curated list of enriched genes from previously published subpopulation data, as described.^88^ Ipsilateral sample enrichment was calculated against the same combined contralateral baseline mentioned above. Gene Ontology (GO) term analyses for differentially expressed genes (DEGs) were performed using the Wallenius method using goSeq (R).^85^ The filtered count data of expressed, non-DEG genes were used as a background. Protein interaction networks were generated using STRING.^70^

#### 2.6.6. Injury signature enrichment

Acute and late injury signatures were calculated using supervised principal component analyses (SPCAs)^6^ on DEGs at 3 days or 4 weeks from the general injury analysis. Eigenvectors were extracted from the first principal component (PC1) and correlated across samples as an unbiased injury signature. For the 4-week timepoint, PC2 was also analysed and loading values were also extracted. These are a product of the covariance between the scaled dimensions and the original variables, giving a weight to how much individual genes contribute to each principal component.

### 2.7. Data accessibility

This data set highlights molecular changes in sensory neuron subtypes across multiple timepoints in a murine neuropathic pain model. To improve the accessibility of these data, an open-source database is available at https://livedataoxford.shinyapps.io/drg-directory/, which includes a shared code to generate personal-omics hosting sites. Raw and processed count data are available on GEO, reference GSE216444.

### 2.8. Tissue staining (immunohistochemistry and in situ)

Adult animals were overdosed with pentobarbital and perfused transcardially with sterile saline followed by 4% paraformaldehyde. Tissue for immunohistochemistry (IHC) was removed and postfixed before subsequent dehydration in 30% sucrose (0.1 M PB) at 4°C for a minimum of 48 hours. Samples were then embedded in OCT medium (Tissue-Tek), sectioned, and stored at −80°C. Neuronal profiles were quantified across multiple sections per animal, opposed to more detailed stereology, and are presented as estimates. In addition to subpopulation markers such as Tyrosine hydroxylase (TH, C-LTMRs), Calcitonin gene-related peptide (CGRP, peptidergic), and parvalbumin (PV, proprioceptors), nonpeptidergic neurons bind isolectin B4 (IB4) from Griffonia simplicifolia. The neurofilament heavy chain (NF200) labels large diameter neurons in mice, and NeuN (or FOX3) is a general neuronal marker.

To validate sex differences in the absence of tamoxifen dosing, fresh DRGs were isolated from littermate pairs of male and female wildtype mice (n = 3, multiple sections per mouse). Tissue was embedded directly in OCT before freezing on dry ice and storage at −80°C. In situ hybridizations (ISHs) were performed using RNAscope Multiplexing v1 and v2 as per the manufacturer's instructions (ACDBio) and TSA Vivid fluorophores (7526/1, 7523/1), with probe details mentioned in Table 3.

**Table 3:** Commercial reagents used in the current study.

Reagent	Source	Identifier	Dilution
Donkey anti-Mouse IgG (H+L), Alexa Fluor 488	ThermoFisher Scientific	A-21202	1/200
Donkey anti-Rabbit IgG (H+L), Alexa Fluor 546	ThermoFisher Scientific	A10040	1/200
Donkey anti-Sheep IgG (H+L), Alexa Fluor 488	ThermoFisher Scientific	A-11015	1/200
Donkey anti-Sheep IgG (H+L), Alexa Fluor 546	ThermoFisher Scientific	A-21098	1/200
Goat anti-Chicken IgY (H+L), Alexa Fluor 546	ThermoFisher Scientific	A-11040	1/200
Goat anti-Rabbit IgG (H+L), Alexa Fluor 488	ThermoFisher Scientific	A-11008	1/200
Goat anti-Rabbit IgG (H+L), Alexa Fluor 546	ThermoFisher Scientific	A-11010	1/200
Goat anti-Rabbit IgG (H+L), Pacific Blue	ThermoFisher Scientific	P-10994	1/200
Streptavidin, Pacific Blue conjugate	ThermoFisher Scientific	S11222	1/100
anti-CGRP, rabbit	Peninsular Labs	T4032	1/500
anti-CGRP, sheep	Enzo	Ca1137	1/250-1/500
anti-Glutamine synthetase (GS), rabbit	Sigma-Aldrich	G2781	1/500
anti-NeuN, chicken	Merck Millipore	Abn91	1/500
anti-NeuN, rabbit	Abcam	Ab177487	1/500
anti-NF200, mouse	Sigma-Aldrich	N0142	1/250
anti-NF200, rabbit	Merck Millipore	ABN76	1/1000
anti-Parvalbumin (PV), guinea pig	Frontier Institute	Af1000	1/200-1/500
anti-Tyrosine Hydroxylase (TH), sheep	Merck Millipore	Ab1542	1/400
IB4, streptavidin conjugated	Sigma-Aldrich	L2140	1/100
mm-Ntrk2-C2	ACDBio	423611-C2	1/50
mm-Kcnj11	ACDBio	431451	NA
mm-Kcns1	ACDBio	525941	NA
mm-Cacng2	ACDBio	437221	NA
RNAscope Multiplex Fluorescent Reagent Kit v1	ACDBio	320293	NA
RNAscope Multiplex Fluorescent Reagent Kit v2	ACDBio	323100	NA
TSA Vivid Fluorophore kit 570	Biotechne	7526/1	1/1500
TSA Vivid Fluorophore kit 520	Biotechne	7523/1	1/1500

## 3. Results

**Figure 1.**
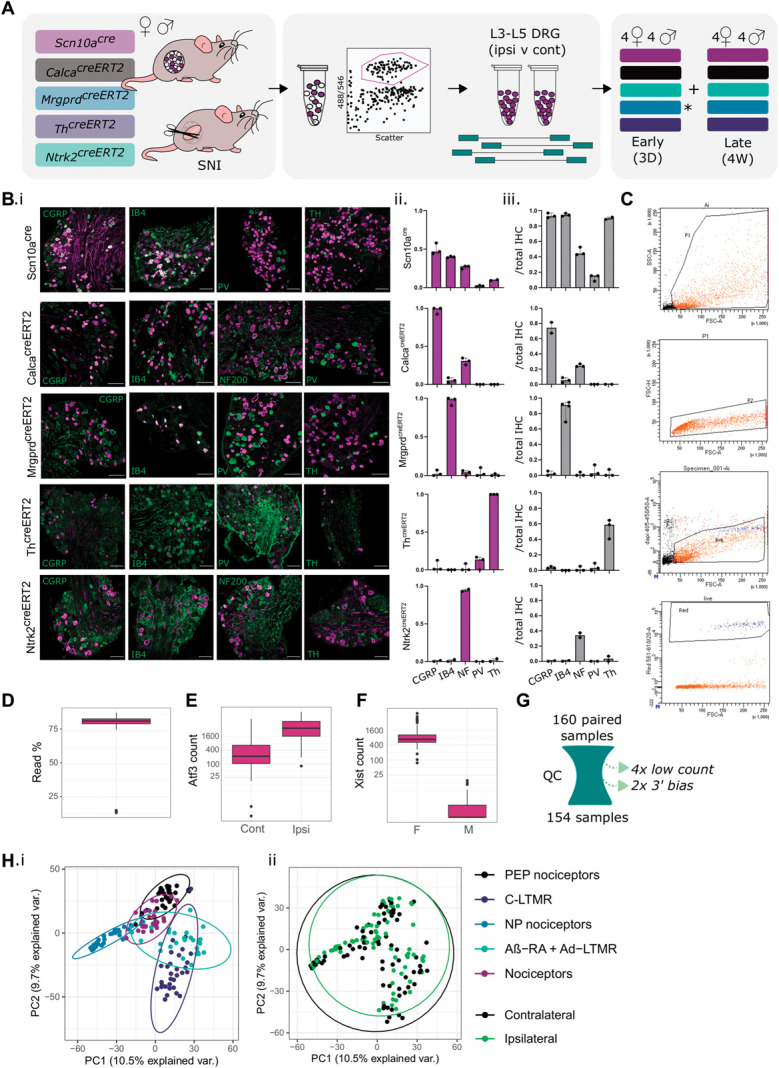
Experimental overview for mouse subtype RNA-seq of 5 neuronal subtypes after nerve injury. (A) Overview schematic, highlighting 5 transgenic mouse lines used to label and sort “bulk” subtype samples for downstream sequencing. Males and females were collected 3 days (3D) and 4 weeks (4W) after spared nerve injury (SNI). (B) Transgenic validation of Scn10a^cre^, Calca^creERT2^, Mrgprd^creERT2^, Th^creERT2^, and Ntrk2^creERT2^ lines. (B.i) Example IHC. (B.ii) IHC overlap with reporter line. (B.iii) Reporter overlap with IHC. (C) Samples FACS gating (*Mrgprd*+ cells, gating for scatter, live/dead, and tdTomato), with addition details in Supplemental Figure 1, available at http://links.lww.com/PAIN/B823. (D) Percentage of uniquely mapped reads by sample. (E) *Atf3* raw count data. (F) *Xist* raw count data. (G) Schematic of QC. 154 samples passed. (H) PCA biplot by subtype (i) and injury status (ii). Plots uncorrected for batch are shown in Supplemental Figure 2, available at http://links.lww.com/PAIN/B823. C-LTMR, C low-threshold mechanoreceptor; DRG, dorsal root ganglia; IB4, isolectin B4; PCA, principal component analysis; PEP, peptidergic; PV, parvalbumin; QC, quality control.

Using transgenic labelling of neuronal DRG subtypes, 160 lumbar DRG samples were sequenced 3 days and 4 weeks after SNI (Figs. 1A–H, Supplemental Figures 1A–L and 2A–D, available at http://links.lww.com/PAIN/B823). This includes 5 neuronal subtypes sorted by fluorescence: general nociceptors, encoded by Scn10^cre^ (nociceptors); peptidergic nociceptors from Calca^creERT2^ (PEP/peptidergic); nonpeptidergic nociceptors by Mrgprd^creERT2^ (NP/nonpeptidergic); C-low threshold mechanoreceptors encoded by Th^creERT2^ (C-LTMRs); and Ntrk2^creERT2^ expressing LTMRs (rapidly adapting (Aβ-RA) and D-Hairs (Aδ-LTMRs)) (Figs. 1B–C, Supplemental Figure 2, available at http://links.lww.com/PAIN/B823). We recognize that our general nociceptor population expressing *Scn10a* does not exclusively comprise high threshold afferents. C-LTMRs are included within this subtype, based on the coexpression of *Th* and *Scn10a* (Fig. 1B).^45^ They make up a much smaller proportion of overall cells than the peptidergic and nonpeptidergic nociceptor subpopulations, resulting in a “nociceptor-like” population.

Together, 154 samples passed QC, removing samples with low read counts or 3′ bias. Male and female samples are clearly distinguishable by sex-linked genes such as *Xist*, and ipsilateral (“injured”) samples can be distinguished from contralateral controls by key injury markers such as *Atf3* (Figs. 1D–G). A batch effect was introduced on the first sample collection day, affecting paired (ipsilateral and contralateral) samples for Calca^creERT2^ and Scn10a^cre^ females (Supplemental Figure 2, available at http://links.lww.com/PAIN/B823). We controlled for this effect in all downstream analyses (see methods).

Sensory neurons undergo broad, stereotyped changes after injury.^51^ Even so, samples largely cluster by neuronal subtype across conditions (Fig. 1H). Although the bulk subpopulation methodology used here allows deep sequencing within populations, each resulting sample contains a mix of injured and intact neurons from ipsilateral ganglia. This cell mixture likely dampens the stereotyped changes seen previously in single-cell RNA-seq.^51^

Analyses were first performed on contralateral (“naïve”) samples. As expected, samples initially cluster by batch before clustering by subpopulation (Supplemental Figures 2C–D, available at http://links.lww.com/PAIN/B823). General nociceptors (nociceptors) as well as peptidergic and nonpeptidergic nociceptor subpopulations largely separate from C-LTMRs and *Ntrk2*-labelled Aβ-RA + Aδ-LTMRs.

### 3.1. Contralateral (“naïve”) samples match previous data

**Figure 2.**
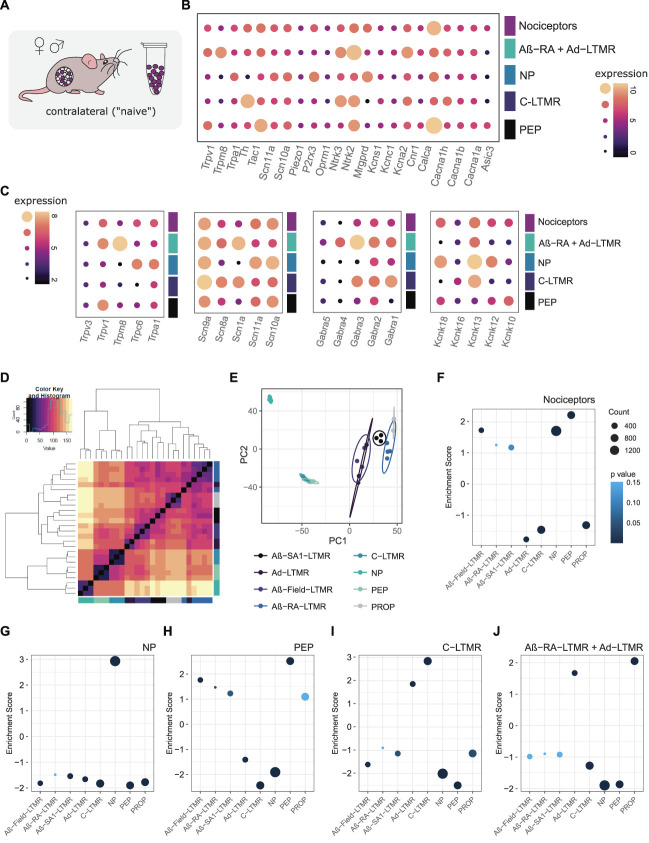
RNA-seq validation against previously published work, combining male + female samples. (A) Contralateral tissue was compared with previously published naïve data sets. (B) Hallmark gene expression across contralateral samples. Expression plotted as VST transformed count data. (C) Ion channel expression across contralateral samples. (D and E) Zheng et al. 2019 naïve subpopulation clustering (mixed sex). (F–J) Subtype enrichment against gene sets derived from Zheng et al. 2019 (see methods for details, gene sets provided in Supplemental Digital Content, Table 3, available at http://links.lww.com/PAIN/B826). (F) Nociceptors, (G) peptidergic nociceptors, (H) nonpeptidergic nociceptors, (I) C-LTMRs, and (J) Aβ-RA + Aδ-LTMRs. Plotted as normalized enrichment scores, coloured by *P*-value. Full lists of scores and *P*-values are available in Supplemental Digital Content, Table 2, available at http://links.lww.com/PAIN/B825. C-LTMRs, C low-threshold mechanoreceptors; NP, nonpeptidergic; PEP, peptidergic; PROP, proprioceptors; VST, variance stabilizing transformation.

In line with previous reports, hallmark gene expression can be seen within each population (Fig. 2A-C, Supplemental Digital Content, Table 1, available at http://links.lww.com/PAIN/B824). Voltage-gated sodium (*Scn*) channels, transient receptor potential (*Trp*), Gamma-aminobutyric acid (*GABA*) receptors (*Gabra*), and 2 pore potassium channels (*Kcnk*) show varying subtype specificity (2B-C).

*Scn10a* and *Scn11a* are enriched in high threshold populations, whereas *Scn1a* is enriched in LTMRs. *Mrgprd* and *Mrgpr* family members, *P2r x3*, *Pirt*, and *Trpa1*, are enriched in nonpeptidergic nociceptors, in line with previous reports. *Ntrk2*, *Scn1a*, and *Trpc1* are enriched in Aβ-RA + Aδ-LTMRs, whereas *Th*, *Tafa4*, *Gfra2*, and *Slc17a8* (VGLUT3) are all enriched in C-LTMRs. Key peptidergic markers such as *Calca* and *Trpv1* do not hit our enrichment filtering criteria, likely due to strong expression in both peptidergic and general nociceptor populations.

Trpm8, typically a marker of cold-sensing sensory neurons,^71^ is enriched in Aβ-RA + Aδ-LTMRs, with lower expression in peptidergic nociceptors (Fig. 2B). This contrasts reports of previous subpopulation RNA-seq, suggesting enrichment in C-LTMRs, peptidergic nociceptors, and Aδ -LTMRs.^88^ It is unclear if this discrepancy reflects a variation across transgenic approaches (eg, a bias towards Aδ -LTMRs), individual animal differences, or is a result of using contralateral samples as “naïve-like” controls. Two-pore potassium channels (K2Ps) have been implicated in various pain conditions, including the role of *Kcnk18* (TRESK) in migraine.^37,62^ We see a range of subpopulation enrichments for this gene family in our data set. *Kcnk13* (THIK-1), *Kcnk12* (THIK-2), and *Kcnk18* (TRESK) are all enriched in nonpeptidergic nociceptors. *Kcnk16* (TALK-1) is enriched in C-LTMRs. *Kcnk10*, encoding the TREK-2, has previously been implicated in spontaneous pain in rats, with expression limited to IB4-binding neurons.^1^ Here, we see higher expression in peptidergic nociceptors. This peptidergic-enrichment profile is supported by the transcriptional data published by Zheng et al.^88^

To validate our sequencing approach, gene enrichment analyses were performed against previously published naïve subtypes (Figs. 2D–J, Supplemental Digital Content, Tables 2 and 3, available at http://links.lww.com/PAIN/B825 and http://links.lww.com/PAIN/B826). Count data were log transformed using DESeq2, mirroring our analysis to generate 8 subpopulation-specific groups, as defined by Zheng et al.^88^

Our samples correlate strongly to this these. Our *Scn10a* population seems to be largely nociceptor, with positive enrichment for both PEP and NP populations (Fig. 2F). Negative enrichment is seen for C-LTMRs, likely reflecting subpopulation proportions within this broad grouping. Our NP samples are positively enriched for the NP gene set and negatively enriched for all other gene signatures (Fig. 2G). To complement, our PEP samples show strong positive enrichment for PEP, as well as negative enrichment for NP and C-LTMR signatures (Fig. 2H). Unlike our peptidergic population, our general nociceptor is negatively enriched for the proprioceptive signature. Both our nociceptor and peptidergic nociceptor populations show negative enrichment for the Aδ-LTMR gene set but positive enrichment of Aβ-Field-LTMR signatures.

C-low threshold mechanoreceptors are highly enriched for the C-LTMR gene set, along with a positive enrichment of Aδ-LTMRs (Fig. 2I). Correspondingly, this population shows negative enrichment of high-threshold populations (NP and PEP), along with the proprioceptive and Aβ-Field-LTMR signatures.

Aβ-RA + Aδ-LTMRs show positive enrichment for the proprioceptive gene set, followed by enrichment for Aδ-LTMRs (2J). As expected, this population is negatively enriched for high threshold signatures. Enrichment scores and adjusted *P*-values are listed in Supplemental Digital Content, Table 2, available at http://links.lww.com/PAIN/B825.

We next compared our data against recently published transcriptomic and proteomic data from Nav1.8-enriched male samples (Supplemental Figure 3, available at http://links.lww.com/PAIN/B823). Here, researchers used the DTA ablation of *Scn10a* neurons in mice to probe enrichment/depletion in nociceptors.^64^ The transcriptomic data for their enriched and depleted genes matches our subpopulation data with enrichment for nociceptors, NP, and CGRP paired to a negative enrichment for our LTMR populations (Supplemental Figure 3A, available at http://links.lww.com/PAIN/B823).

We were also able to recapitulate their proteome enrichment for our nociceptor and NP populations, but not PEP (no significant enrichment), whereas the Aβ-RA + Aδ-LTMRs population is negatively enriched (Supplemental Figure 3A, available at http://links.lww.com/PAIN/B823). With few depleted proteins, we are less powered to analyse depleted targets. Using more relaxed thresholds (FDR < 0.05, LFC > 0.5, Supplemental Figure 3B, available at http://links.lww.com/PAIN/B823), the trend remains unclear, likely due to the discrepancy between transcriptomic and proteomic data^8,25^ as well as differences in methodology. Together, these enrichments lend confidence to our methodology and support the use of this data set to interrogate population sex differences and as a baseline against injured neurons.

### 3.2. Naïve sex differences across subtypes

**Figure 3.**
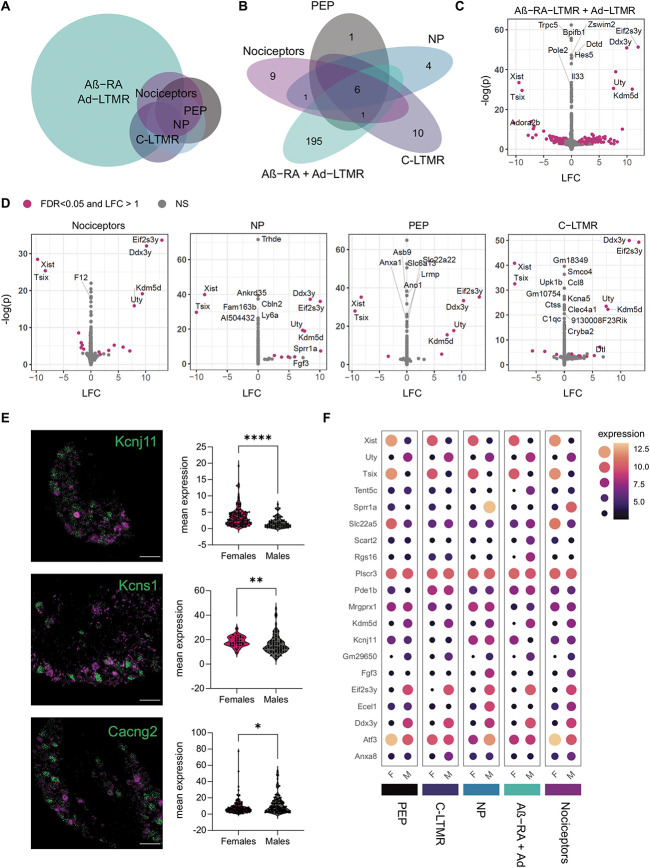
Few sex differences are seen in uninjured neuronal subtypes, with the majority in *Ntrk2*+ LTMRs. (A) Euler plot for sexually dimorphic genes. (B) Number of sexually dimorphic genes within each subpopulation examined (FDR < 0.05, LFC > 1). (C and D) Contralateral samples show differential gene expression across sexes (male vs female, ie, LFC > 0 = upregulated in males). (E) In situ validation of gene candidates regulated in Aβ-RA + Aδ-LTMRs (n = 3 mice, Mann–Whitney test. Data points represent individual cells). Top to bottom: *Kcnj11* (*P* < 0.0001), *Kcns1* (*P* = 0.0054), and *Cacng2* (*P* = 0.0242) (green) with *Ntrk2* (magenta). *Cacng2* RNA-seq suggests upregulation in females (opposite). (F) Dot plots highlighting key DEGs, plotted as median transformed counts. DEGs, differentially expressed genes; LFC, log_2_ fold changes; LTMRs, low=threshold mechanoreceptors; NP, nonpeptidergic; PEP, peptidergic.

With many clinically relevant pain conditions showing sexual dimorphisms, there is a keen interest to explore sex differences within each DRG subtype transcriptome. Across subpopulations, most genes are expressed to similar levels in males and females (Figs. 3A–D). Differentially expressed genes are defined here as an FDR < 0.05 and an absolute LFC > 1. From this, only 6 genes were significantly regulated between males and females in all populations, each X-linked or Y-linked (*Kdm5d*, *Uty*, *Ddx3y*, *Eif2s3y*, *Tsix*, and *Xist*). *Gm29650*, which is also sex-linked, was differentially expressed in 4/5 populations. The only other DEG shared across any subtypes is *Sprr1a*, which is more highly expressed in male nociceptors and nonpeptidergic nociceptors, and may reflect differences in naïve cells or minor wounds from in-cage fighting which occur at higher rates in males.

This absence of a large sensory-neuron wide sex signature is consistent with the previous work in mice, where all but one DEG reported in adult, lumbar DRG were X-linked or Y-linked.^66^ Nine autosomal genes were found to be regulated in sacral DRG, and these do not overlap with the DEGs reported within populations here, with the exception of *Clvs1* in Aβ-RA + Aδ-LTMRs.

At a subpopulation level, a stronger sexual dimorphism emerges. Across populations, many genes hit an FDR < 0.05, but moderated fold changes suggest a negligible effect (near 0 LFC) in these genes (Figs. 3A–F, Supplemental Digital Content, Tables 4 and 5, available at http://links.lww.com/PAIN/B827 and http://links.lww.com/PAIN/B828). Most DEGs are seen in lowly expressed genes within unique neuronal subtypes. We see predominant transcriptional changes within Aβ-RA + Aδ-LTMRs (202 genes with FDR < 0.05, LFC > 1), with few differences in the other populations. Here, RNA-seq and in situ hybridization validation of *Kcnj11* and *Kcns1* (Fig. 3E) suggest they are more strongly expressed in female TRKB+ LTMRs. *KCNS1* has previously been suggested as a marker of LTMRs in humans,^72^ and cross-species validation of possible sexual dimorphism in TRKB+ and other LTMR populations is recommended. Not all candidates were validated using in situ by naïve, wildtype controls. Of the 3 candidates explored, *Cacng2* (Stargazin) is significantly upregulated in males by in situ (3E) but downregulated in our RNA-seq results (Supplemental Digital Content, Table 4, available at http://links.lww.com/PAIN/B827). This still suggests sexual dimorphism within this LTMR population but warrants note as it may be a result of population labelling through in situ vs transgenics or a difference in contralateral vs naïve tissue.

GO term analyses highlight enrichment for ion channel transport and transmembrane transport in females, although few genes are implicated in each (Supplemental Digital Content, Table 6, available at http://links.lww.com/PAIN/B829). GSEA analyses against “all gene sets” available from Molecular Signatures Database (MSigDB) show no enrichments in this population (Supplemental Digital Content, Table 7, available at http://links.lww.com/PAIN/B830).

In the 4 other populations studied, most GO terms centre around sex-linked processes. Other relevant GO pathways include the detection of temperature stimulus involved in sensory perception of pain (nociceptors), immune response and cholinergic synaptic transmission (PEP), regulation of sensory perception of pain (NP), and chemosensory behavior (C-LTMR). All Supplemental tables for DEGs and pathway analyses, including full DEG tables, GO, and GSEA analyses for each subpopulations, are available in the supplemental digital contents.

### 3.3. General injury signatures

**Figure 4.**
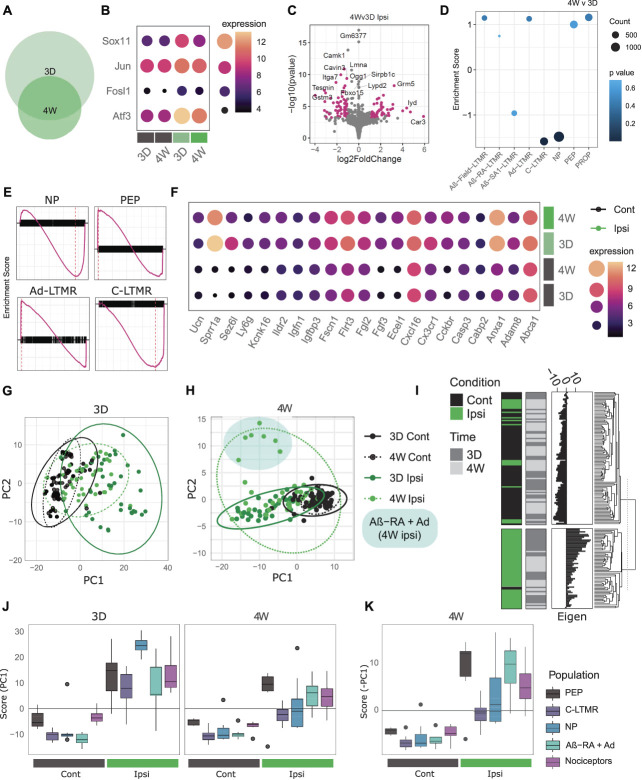
General injury mapping shows stereotyped changes and subtype differences across time points. (A) Euler plot showing differentially expressed genes after SNI at 3 days (3D) and 4 weeks (4W) for combined subtypes. (B) Key injury markers are upregulated across ipsilateral samples at both time points, plotted as median VST expression across groups. (C) Injured samples (ipsi) show differential gene expression across time points (4W vs 3D). (D and E) GSEA analysis of subtype enrichment between 4W and 3D ipsilateral samples. NP and C-LTMR signatures are significantly reduced at 4 weeks, with values listed in Supplemental Digital Content, Table 12, available at http://links.lww.com/PAIN/B835. (E) Enrichment plots for key subtypes, by the enrichment score for ranked genes (black). (F) Example of differentially expressed genes (DEGs) shared across time points, plotted as median VST expression across groups. (G and H) Supervised PCA biplot for DEGs at 3 days (G) and 4 weeks (H). (I) Dendrogram split by k-means of 2, highlighting positive and negative injury scores from 3D DEGs largely correlate to the sample condition. (J and K) PC1 correlation across subtypes and time. Boxplot whiskers show 1.5 IQR. (J) 3D signature: All 5 subtypes show a significant difference between ipsilateral and contralateral samples at both time points (Kruskal–Wallis rank sum, followed by pairwise Wilcoxon with BH correction against a grouping factor [population, condition, and timepoint]). Only NP and C-LTMR ipsilateral samples are different between 4 weeks and 3 days (FDR = 0.00185 and 0.00995, respectively), reflecting their return towards baseline. (K) 4W signature: All 5 subtypes show a significant difference between ipsilateral and contralateral samples, plotted as score for visualization with (J). Cont, contralateral; Ipsi, ipsilateral; IQR, interquartile range; LTMR, low-threshold mechanoreceptors; NP, nonpeptidergic; PCA, principal component analysis; PEP, peptidergic; SNI, spared nerve injury; VST, variance stabilizing transformation.

General injury signatures were examined by combining samples across subtypes (Figs. 4A–K, Supplemental Digital Content, Tables 8–12, available at http://links.lww.com/PAIN/B831, http://links.lww.com/PAIN/B832, http://links.lww.com/PAIN/B833, http://links.lww.com/PAIN/B834, http://links.lww.com/PAIN/B835). These samples will be biased toward nociceptors, due to the inclusion of 3 nociceptor populations (Scn10a^cre^, Calca^creERT2^, and Mrgprd^creERT2^) and 2 LTMR populations (Th^creERT2^ and Ntrk2^creERT2^) in equal numbers.

At both 3 days and 4 weeks, we see predominant upregulation of genes associated with classical injury signatures, including *Atf3*, *Jun*, *Sox11*, and *Fosl1* (Fig. 4B, Supplemental Digital Content, Table 8 http://links.lww.com/PAIN/B831). The overall number of DEGs (LFC > 1, FDR < 0.05) is reduced over time, from 521 at 3 days to 162 by 4 weeks. Of these, 96 are shared across time points (Supplemental Digital Content, Table 9 http://links.lww.com/PAIN/B832), with some highlighted in Figure 4C. Subtype-enriched genes seem acutely downregulated at 3 days, in line with previous reports although few genes reach our significance threshold. This is likely due to the high variability from combining samples across populations.

Our time course was selected to highlight the progression from a more acute to a later injury state after SNI (Figs. 4C–D). We can probe this transition by comparing ipsilateral samples across time points. At 4 weeks, we see the downregulation of *Atf3*, as well as an upregulation of some subtype-specific genes towards baseline, such as *Ntrk2*. *Slc17a7* (VGLUT1), typically a marker of larger diameter DRG is positively enriched at 4 weeks compared with 3 days after SNI (LFC = 2.25, p.adj = 1.7E-07), although this change is not reflected by a median expression change and is likely driven by a subgroup of samples. For other genes, such as *Npy*, gene expression increases from 3 days to 4 weeks, where it is significantly higher than contralateral levels, suggesting a more long-term change.

In a more acute state, GO analyses show enrichment in the regulation of cell population proliferation, positive regulation of apoptotic process, and inflammatory response after injury (Supplemental Digital Content, Table 10, http://links.lww.com/PAIN/B833). Many of these processes remain enriched at 4 weeks, even with the overall reduction in DEGs. GSEA enrichment at 3 days also shows downregulation of electron transport and oxidative phosphorylation paired to a positive enrichment of inflammation, receptor regulator activity, and cell migration (Supplemental Digital Content, Table 11, available at http://links.lww.com/PAIN/B834). When comparing ipsilateral samples over time, GO analyses also suggest functional changes. Three-day injured samples show enrichment of apoptotic process, cytokine response, and positive regulation of gene expression. In a later state, there is enrichment for protein import, long-term memory, and the regulation of long-term neuronal synaptic plasticity. Taken together, these results suggest we are accurately capturing injury signatures across our data set.

### 3.4. Injury phenotypes by subtype

**Figure 5.**
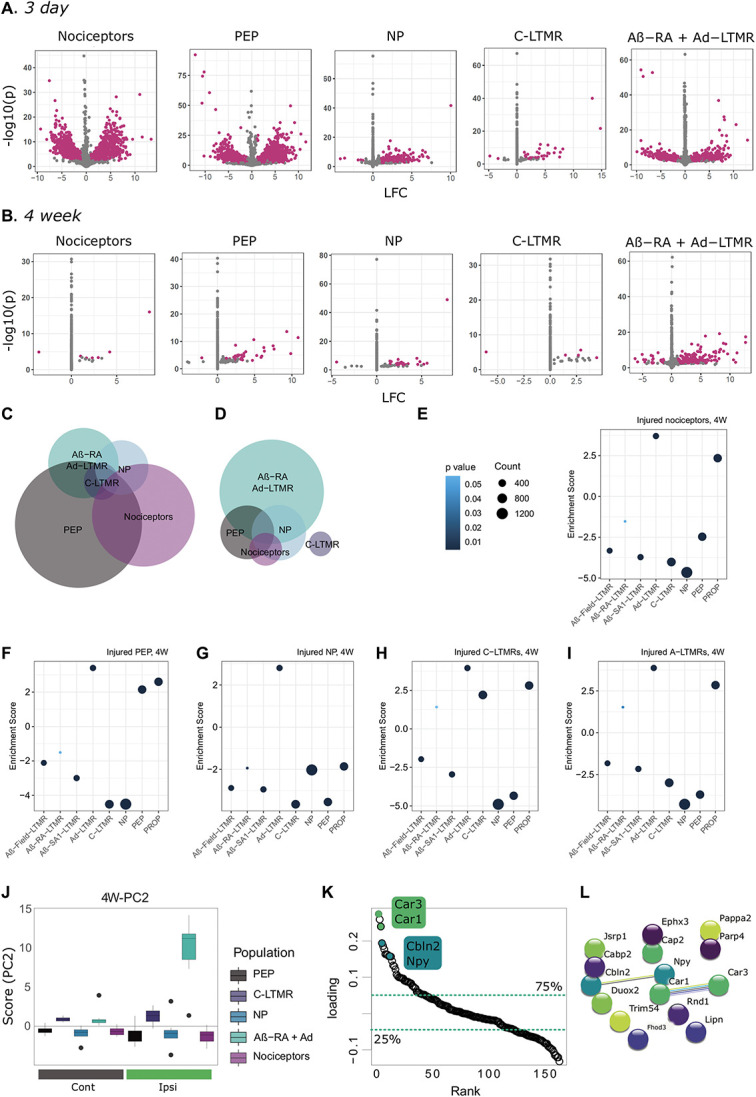
Subtype injury mapping shows stereotyped changes and subtype differences across time points. (A–D) Differential gene expression across time points and subtypes. Volcano plots for 3-day (A) and 4-week (B) subtypes, with DEGs highlighted in magenta. Euler plots showing DEG overlap at 3D (C) and 4W (D). (E–I) GSEA analyses against previously published data^88^ reveals a lack of clear subpopulation signatures in injured nociceptors and NP nociceptors by 4 weeks (scores listed in Supplemental Digital Content, Table 18, available at http://links.lww.com/PAIN/B841). All subtypes show a significant enrichment for Aδ-LTMRs, which was not seen in naïve nociceptor populations. Naïve data are shown in Figures 2J,L: Subtype-specific signatures extracted from 4W SPCA presented in Figure 4 shows specificity for Ntrk2-injured neurons. (J) PC2 correlation across subtypes 4 weeks after SNI. Boxplot whiskers show 1.5 IQR. (K) Ranked loadings (PC2) for all DEGs at 4 weeks after injury. Dashed lines highlight quartiles. L. STRING database interactions for top 15 DEGs (ranked by loadings). DEGs, differentially expressed genes; IQR, interquartile range; LFC, log_2_ fold changes; LTMR, low-threshold mechanoreceptors; NP, nonpeptidergic; PEP, peptidergic; PROP, proprioceptors; SNI, spared nerve injury; SPCA, supervised principal component analysis.

A major strength of this study is the ability to probe subtype specific patterns in a murine model of neuropathic pain. Injured neurons were previously shown to lose cell-type specific identities after nerve injury in a time-dependant process.^60^ At 4 weeks post-SNI, injured samples show a negative enrichment for C-LTMRs and NP nociceptors compared with their 3-day injured counterparts (Figs. 4D–E). Here, we are also able to explore subpopulation-specific and common injury signatures across cell types (Figs. 4G–K), before contrasting samples within each population (Fig. 5).

To start, an acute injury signature was extracted from our initial list of DEGs at 3 days (from combined samples, above) through a supervised PCA (SPCA) and compared across subtypes (Figs. 4G,I–J). This provides an unbiased signature through the linear combination of individual gene expressions. All 5 subtypes studied show a significant difference (FDR < 0.05) between ipsilateral and contralateral samples at both time points (Kruskal–Wallis Rank Sum, followed by pairwise Wilcoxon with Benjamini-Hochberg (BH) correction against a grouping factor [population, condition, and timepoint]). Only NP and C-LTMR show a significant difference between 4 weeks and 3 days (FDR = 0.00185 and 0.00995, respectively), reflecting their stronger return towards baseline.

Eigenvectors were also extracted from an SPCA on 4W DEGs to form a late injury signature across populations (Fig. 4H). This signature is driven largely by general nociceptors, peptidergic nociceptors, and Aβ-RA + Aδ-LTMRs, although all subtypes show a significant difference from their contralateral counterparts at 4 weeks (Kruskal–Wallis Rank Sum, followed by pairwise Wilcoxon with BH correction) (Fig. 4K).

### 3.5. Differential gene expression changes by subtype

Within population testing for differentially expressed genes show a number of shared regulated genes typical of injury signatures (including *Atf3*, *Sprr1a*, and *Sox11*), as well as a subset of subtype-specific DEGs (Figs. 5A–D, Supplemental Digital Content, Tables 13–18, available at http://links.lww.com/PAIN/B836, http://links.lww.com/PAIN/B837, http://links.lww.com/PAIN/B838, http://links.lww.com/PAIN/B839, http://links.lww.com/PAIN/B840, http://links.lww.com/PAIN/B841). Over 5000 genes are regulated overall and are mostly driven by changes in the general nociceptors (2620) and peptidergic nociceptor (3270) samples at 3 days. Fewer DEGs are seen in other populations, with 179 DEGs for nonpeptidergic nociceptors, 640 DEGs for Aβ-RA + Aδ-LTMRs, and only 36 DEGs in C-LTMRs (with an LFC > 1, FDR < 0.05). Using an LFC cutoff of 1, we are focusing on genes with good expression changes believed to correspond to biological relevance. Even so, small changes in gene expression can be biologically relevant, and full results tables are provided online. With the populations studied, we do not see a clear LTMR-specific pattern: No genes exclusively regulated in both C-LTMRs and Aβ-RA + Aδ-LTMRs.

Although there is a broad diversity in genes regulated, we also see patterns at a group level. For example, we see broad dysregulation of potassium channels—a channel grouping widely implicated in pain.^12,75^ In nociceptors, changes predominate at 3 days, with an upregulation of numerous potassium channel genes, including *Kcnk16* and *Kcnh6*, paired to a downregulation of others (*Kcna6, Kcnb1, Kcnd1, Kcnd3, Kcnh2, and Kcnh6*). In PEP, we see similar patterns with an upregulation in various K-channels (eg, *Kcnk13, Kcnk16, Kcnj2, Kcnj11, and Kcns3*) and a downregulation in others (*Kcnn2, Kcnj16, and Kcnh2*). This effect is not limited to nociceptor populations. We see further regulation in Aβ-RA + Aδ-LTMRs: 3 days after SNI, *Kcnq5, Kcnk18, Kcnk10,* and *Kcnt1* are all downregulated.

A number of these candidates fit the previous literature. For example, in Aβ-RA + Aδ-LTMRs, *Kcnq5* mediates M currents which have been linked to pain,^56^ *Kcnk18* (TRESK) a 2-pore potassium channel that has been described for its role in migraine as well as DRG hyperexcitability,^82^ and *Kcnt1* (SLACK) has been described in the context of neuropathic pain after SNI.^39^ Most genes are either regulated in the same direction or only regulated in a subset of populations. Even so, over 400 DEGs are regulated in opposing directions, which may provide a unique look at subtype differences after injury (Supplemental Digital Content, Table 14, available at http://links.lww.com/PAIN/B837). For example, *Ints5* is an integrator complex involved in RNA transcription which is upregulated in PEP and general nociceptors while being downregulated in Aβ-RA + Aδ-LTMRs. The GTP binding protein *Gtpbp1* has been previously implicated in neuronal death through translational regulation.^73^ Here, we see subtype differences in regulation, being upregulated in PEP and Aβ-RA + Aδ-LTMRs but downregulated in general nociceptors (with downward trends in NP and C-LTMRs). Other genes showing bidirectional regulation include *Rnd1*, or Rho Family GTPase 1, which has been discussed previously for its role in axon outgrowth, whereas *Wnk4* is related to actin cytoskeleton remodelling by Rho GTPases, as well as ion channel regulation.

At 4 weeks post-SNI, there is a reduced number of DEGs across all subtypes, compared with our 3-day results (Fig. 5B). The most changes are present in Aβ-RA + Aδ-LTMRs (217 DEGs). Nonpeptidergic and peptidergic nociceptors show the next highest number of DEGs, with 25 and 36, respectively (sharing *Cckbr*, *Tubb6*, *Atf3*, *Gpr151*, *Wt1*, *Pde6b*, *Cyp26a1*, and *Cdk6*). Few changes are seen in our general nociceptor population, with only 7 DEGs with a moderated LFC > 1 (*Cckbr*, *Ttll10*, *Phox2b*, *Rpl31-ps13*, *S100a8*, *Gata5os*, and *Gm47138*). C-LTMRs also show few changes (5 DEGs), in line with the acute signature (*Hba-a1*, *Nefh*, *S100b*, *Adtrp*, and *Gm35097*). No genes show regulation in opposing directions across subtypes by 4 weeks, although some, such as *Rnd1*, remain regulated.

Many studies highlight cell-type or injury-specific gene regulation mechanisms, as they regulate important mechanisms of neuropathic pain. In addition, they provide possible new avenues to target and modulate neurons in injured states. Our current data set is well suited for these enquires. Across time points, a number of DEGs correspond to transcription factors (“GO:0003700”) or are involved in gene regulation (“GO:0010468”). This includes shared regulators such as *Atf3*, *Hoxa2*, *Twist2*, *Cdk6*, *Prdm10*, and *Trim34b* as well as numerous subtype-specific regulators (Supplemental Digital Content, Tables 16 and 17, available at http://links.lww.com/PAIN/B839, http://links.lww.com/PAIN/B840).

Next, we queried cell type enrichment in our injured samples (Figs. 5E–I, Supplemental Digital Content, Table 18, http://links.lww.com/PAIN/B841). By 4 weeks post-SNI, GSEA enrichment of subpopulation signatures varies from their naïve counterparts, previously discussed in Figure 2. Injured nociceptors and NP nociceptors no longer show clear subpopulation delineations, whereas injured PEP and C-LTMRs both show enrichment for their respective populations. Across all subtypes, there is a positive enrichment in the Aδ-LTMR signature, previously only seen in naïve LTMR samples. Injured Aβ-RA + Aδ-LTMRs also show a new negative enrichment for Aβ-Field and Aβ-SA1-LTMRs.

Using a general injury signature in Figure 4, a subset of samples shared variation in PC2. This eigenvector was also extracted for comparison (Figs. 5J–L). Here, this variation is driven by Aβ-RA + Aδ-LTMRs ipsilateral samples, suggesting a distinct injury signature in this population not captured in the other subtypes. Gene loadings for PC2 were extracted and ranked by loading for further analyses (5K), with the top and bottom 25% quartiles extracted for GO analyses. Both “response to stimulus” and “actin filament organization” were upregulated GO terms, whereas “immune response” and “synapse maturation” were downregulated. No GSEA enrichments on ranked loadings were present.

The STRING database was next queried for possible interactions between gene products. In the top quartile (41 DEGs), interactions are seen between *Car1-Car3*, *Unc-Npy-Cbln2*, *Hrk-Pmaip1*, and *Uox-Mbl2*. Interactions for the genes with the highest loadings are highlighted in Figure 5L. Neuropeptide Y (*Npy*) and cerebellin-2 (*Cbln2*) have previously been implicated in mechanical hypersensitivity.^63^ *Npy* has also been well documented for its upregulation in large-diameter neurons after injury, which may explain the subtype-specific effect seen here.^79^

### 3.6. Sexual dimorphism in injured states

**Figure 6.**
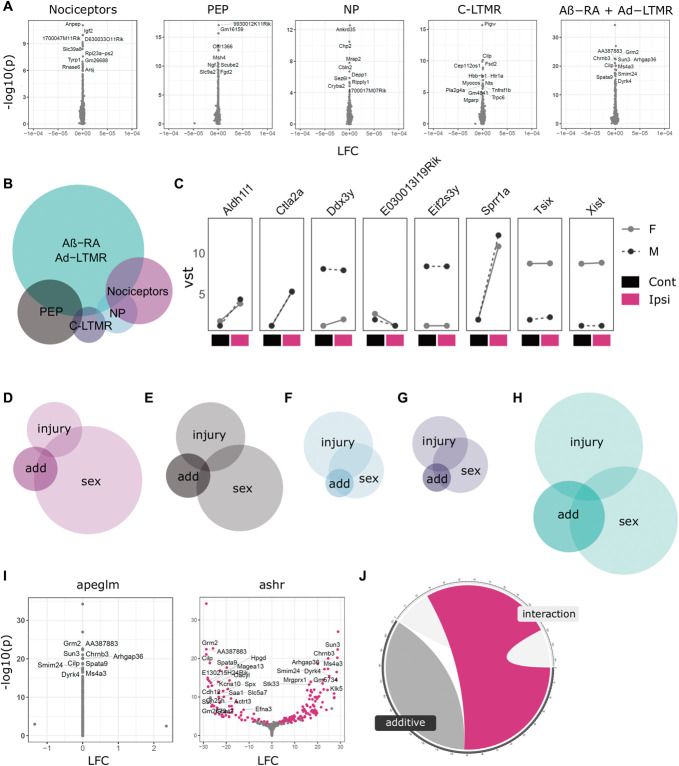
Sexual dimorphism in neuronal subtype injury responses. (A) Transcriptomic analyses in primary afferents reveal no clear interaction of sex and injury 4 weeks after SNI. (B) Euler plot of DEGs using an additive model contrasting sex and injury differences. (C) Line plots of DEGs shared across at least 2 subtypes. (D–H) Across subtypes, DEGs from this additive modelling (“add”) appear to be driven partly by sex differences in basal expression levels (“sex”), as well as some overlap with genes generally regulated in injury (“injury”). (D) General nociceptors. (E) PEP nociceptors. (F) NP nociceptors. (G) C-LTMRs. (H) Aβ-RA + Aδ-LTMRs. (I) Example volcano plots for the interaction of sex and injury for Aβ-RA + Aδ-LTMRs, with *apeglm* and *ashr* shrinkage. (J) 42% of regulated genes are shared across our interaction and additive models (magenta) which are not regulated with *apeglm*. Cont, contralateral; DEGs, differentially expressed genes; Ipsi, ipsilateral; LFC, log_2_ fold changes; LTMR, low-threshold mechanoreceptors; NP, nonpeptidergic; PEP, peptidergic; SNI, spared nerve injury.

To see if male and female sensory neurons differ in their maintenance of later neuropathic pain states, we fitted an interaction model for sex and condition (Fig. 6). Acute effects were not studied due to the colinearity of the batch and sex in a subset of the populations. Using this stringent modelling, which requires genes to be regulated in injury, as well as have a differential response to sex, we detected no differences when pooling populations at 4 weeks or when subsetting our data to interrogate within subtype (Fig. 6A). The number of genes with FDR < 0.05 range from 9 (NP) to 212 (Aβ-RA + Aδ-LTMRs), but moderated fold changes centre towards zero, suggesting these result from the high variability in low count genes, instead of biologically meaningful differences.

By definition, interaction effects are calculated using the reference mean expression from only one sex. This can mask effects in genes which are lowly expressed in one sex during control states. To overcome this, we further explored sexual dimorphism using a more relaxed additive design, subtracting sex, and injury comparisons to pull out sex differences which cannot be explained by the injury effect alone (Figs. 6B–J, Supplemental Digital Content, Table 19, http://links.lww.com/PAIN/B842).

Using this approach a number of DEGs are seen, with the majority in Aβ-RA + Aδ-LTMRs (144 genes), followed by peptidergic nociceptors (41) and general nociceptor (34) populations (Fig. 6B).

Eight genes show regulation in multiple subtypes. This includes a subgroup of sex-linked genes (eg, *Xist*, *Tsix*, and *Kdm5d*), as well as those regulated in injured states, such as *Sprr1a*. Only one, *Tsix*, shows regulation across all subtypes, but this does not seem to be a strong interaction of sex and injury (Fig. 6C). Across subtypes, DEGs from this differential response to injury seem to be driven partly by sex differences in basal expression levels, as well as some overlap with genes generally regulated in injury.

Aβ-RA + Aδ-LTMRs show the largest number of DEGs. Here, there is a 32% overlap with significantly regulated genes from naïve male vs female samples and a 12% overlap with genes regulated in an injured state (6H). Together, 40% of DEGs overlap with significantly regulated genes for sex in control samples or injury at 4 weeks, based on an FDR < 0.05. This does not account for fold changes, due to differences in shrinkage methods (*apeglm* vs *ashr*, see Methods).

For example, the cholinergic receptor, *Chrna3* (a common marker of silent nociceptors),^58^ is not sexually dimorphic in naïve states. As expected, it is most highly expressed in PEP samples (Supplemental Digital Content, Table 4, http://links.lww.com/PAIN/B827). It is also upregulated after SNI in Aβ-RA + Aδ-LTMRs at 4 weeks from a low baseline expression. Its interaction of sex and injury in Aβ-RA + Aδ-LTMRs (Supplemental Digital Content, Table 19, http://links.lww.com/PAIN/B842) suggests caution in its use as a marker in injured states. The functional relevance of this sexual dimorphism also warrants further study. To see if the difference between our interactive and additive modeling was primarily a caveat of shrinkage priors, our interaction fold changes were recalculated using *ashr*, where 42% of DEGs detected through the additive model overlap (Figs. 6I–J). Shrinkage priors aim to limit noise within a data set, and plotting nontransformed counts for these genes highlights the variability present. Some fold changes seem driven by a single sample per condition, whereas others show stronger trends. This highlights how the variability in lowly expressed genes can limit conclusive analyses. Taken together, we interpret this as a lack of strong sexual dimorphism between male and female subtypes specifically in response to injury by 4 weeks. Using a less stringent analysis for sexual dimorphism, we do detect within subtype differences. These are primarily seen in Aβ-RA + Aδ-LTMRs and are partly driven by baseline sex differences at a subpopulation level, which were initially discussed in Figure 3. The variability in low count genes adds noise to these analyses: Even deeper sequencing and in situ validation may still reveal a clearer interaction or lack-there-of between sex and injury.

## 4. Discussion

The availability of transgenic mice paired to advances in low input RNA-seq has allowed deep sequencing of sensory neuron subpopulations after injury. This permits detection of a large number of DEGs not currently possible through traditional sc/snRNA-seq approaches. Building on previously available naïve data from Zheng et al.,^88^ we are able to explore sexual dimorphism in naïve states as well as changes after SNI at 2 time points. We have further probed sexual dimorphism after injury at a subpopulation level.

Together, we see extensive and compelling changes in neuronal gene expression as a consequence of nerve injury. Some of these findings are actionable, by us but also—we hope—by the community as a whole. We have curated our data into an accessible format to encourage exploration and follow-up studies (see: https://livedataoxford.shinyapps.io/drg-directory/).

### 4.1. Injury signatures

Samples cluster primarily by cell type. By collapsing these subtypes, we can extract general injury signatures that mirror injury signatures seen in previous studies.

This general analysis is enriched for sensory neurons. We anticipate this transcriptional signature to be similar to bulk RNA-seq of MACS-purified neurons, where researchers quantified a “nociceptor” transcriptome based on the isolation of small-diameter neurons.^74^ In naïve states, we see many overlapping genes, including key transcription factors they report to be enriched in their “nociceptor” sample. These include *Pou4f2*, *Myt1*, *Ldb2*, *Isl2*, *Bhlha9*, and *Atf3*, which show varying degrees of cell-type specificity in our data. We have built on this with added data after injury, giving insight to possible nociceptor-enriched transcription factors involved in injury. For example, *Isl2* encodes insulin-related protein 2 and is regulated after injury in our data set. It also shows regulation in human patients with diabetic peripheral neuropathy, suggesting cross-species, cross-model target for future experiments.^29^

To probe differences in acute injury and later states, we examined samples at 3 days and 4 weeks. Across ipsilateral samples, we see a reduction in NP and C-LTMR enrichment more chronically. The population data were pooled for GSEA analyses across time points, so changes in gene signatures can be difficult to interpret. For example, if we are working with the assumption that injured NP cells die after SNI, as suggested in West et al. 2020, and hinted at by the loss of IB4-binding terminals after nerve injury in rat,^5^ the ipsilateral NP samples by 4 weeks are likely to contain primarily intact neurons (opposed to cell bodies from transected afferents). The significant reduction of an NP signature over time may thus result from changes in the intact NP neurons, or a bias in the general nociceptor population, which shows enrichment for both PEP and NP nociceptors in a naïve state. C-LTMRs show a similar pattern to NP, although this population remains understudied in comparison with NP nociceptors. Together, this is an interested area for future follow-up, as cell loss has also been documented in human patients with neuropathic pain.^29^ To address this in further detail, and amplify a major strength of the study, subtype-specific analyses were performed.

### 4.2. Subtype-specific injury changes

Previous work has explored injury signatures in whole DRG and single cells.^44^ We are adding a middle ground of “bulk,” subtype-specific population analyses through deep sequencing of neuronal subtypes post-SNI. Deep sequencing allows us to interrogate genes at a larger dynamic range, including lowly expressed genes, and produces an expression matrix that is less sparse than sc/snRNA-seq.

There are likely to be multiple mechanisms, leading to hyperexcitability and pain. Previous work combining computer modelling and experimental manipulations has shown that there may be multiple routes to sensory neuronal hyperexcitability and that normalising any one ion channel conductance may not be sufficient to treat pain.^59^ Moreover, neurons do not exist in a vacuum. Although the current study specifically probes primary afferent transcriptomes, changes in neuronal connectivity as well as glial and immune infiltration/transcriptional changes will be required for a comprehensive understanding. With this, transcriptional changes in neuronal subtypes remain an important piece of the puzzle. Our sequencing depth permits differential expression testing within subpopulations after injury (Figs. 5A–D). All populations show differential gene expression, but this is primarily driven by general nociceptors and PEP at 3 days and Aβ-RA + Aδ-LTMRs at 4 weeks. We see significant upregulation of multiple injury genes and a significant enrichment of a general injury signature in the ipsilateral samples across all subtypes. This suggests the low number of DEGs in other populations is not an artifact of sequencing intact neurons but instead a biologically relevant signature. A number of these DEGs are involved in gene regulation and may be useful targets for genetic manipulation.

In line with previous reports, we also see a reduction in cell-type specificity within our injured samples (Figs. 5E–I).^51,60^ Primarily, we see a reduced signature in general nociceptors, as well as NP nociceptors, whereas PEP nociceptors still show enrichment for PEP, either from the contribution of intact afferents in the samples, or less change in injured cells.

There is an added enrichment for Aδ-LTMRs across all nociceptor subtypes post-SNI, which remains enriched in LTMR populations. The consequence of this is unclear, with multiple, nonmutually exclusive hypotheses available. For example, do nociceptors develop a more LTMR-like signature? Is this driven by a clear subset of genes? Is this signature simply representative of a more general, undefined, or immature sensory neuron? Do Aδ-LTMRs show a more injured phenotype in naïve states? We cannot conclusively exclude this latter possibility, but key injury genes are not present in the gene set curated from Zheng et al.,^88^ and our contralateral *Ntrk2* samples are negatively enriched for our general injury signature at both time points. Together, this suggests they do not show a strong injury phenotype at baseline.^63,79^

### 4.3. Sexual dimorphism

In a naïve state, we see distinct sexual dimorphism across subtypes (Fig. 3). This does not translate to a strong interaction of sex and injury, as the injury response seems to be consistent across sexes (Fig. 6). With baseline differences in gene expression across sexes, a strong interaction with injury is not required for functionally relevant changes in injured states and using an additive model to contrast sex and injury at 4 weeks, we are able to identify possible gene candidates for further validation (Fig. 6).

Proportionally, Aβ-RA + Aδ-LTMRs show the most DEGs here. As a top hit, *Slit3* is an estrogen-sensitive axonal guidance molecule previously discussed in the context of endometriosis and pelvic pain.^26^ Other DEGs include a range of genes involved in inflammation and immune response (eg, *Ifi211*, *Ctla2a*, and *Tlr4*), cholinergic receptors (*Chrna3* and *Chrnb3*), the transcription factor *Neurog3*, and numerous sex-linked genes. The sensitization of primary afferents^55,57^ and higher order circuitry^7^ in a sex-dependant manner has been well established. The lack of a strong dimorphism at the neuronal level seen in the current study suggests that neurons in isolation are not sufficiently sexually dimorphic and likely depend on intercellular (eg, immune^42^) and endocrine (eg, prolactin^14,55,57^ and oestrogen^17,31^) interactions. This fits the previous work in humans, where only minor differences were detected across neuronal subtypes.^72^ Transcriptional differences in control states may still contribute to painful states due to altered immune or glial interactions, baseline excitability, or differences in higher order circuitry. The minor interaction of sex and injury may then act to amplify or suppress some of these signalling pathways and presents an exciting avenue for further study.

### 4.4. Technical limitations

The dissection and dissociation of neurons can be seen as an acute injury model. Moreover, the sensitivity of sensory neurons forces a trade-off between cell health and increased purity when considering increased FACS cycles. We aimed to minimize these confounds through the use of rapid isolations and paired controls. Validation against previously published hallmark genes, as well as gene enrichment sets from prior sequencing experiments gives confidence to our data set.

In addition, these data do not conclusively highlight an interaction of sex and injury within subtypes. The absence of a strong signature fits previous literature, but nuanced changes in lowly expressed genes may still hold biological relevance. Tamoxifen dosing introduces a confound so candidates were validated on naïve, wildtype tissue, and the variability in low count genes makes this difficult to probe with our sequencing depth. Shrinkage methods differ in their LFC estimates at this point, with a conservative shrinkage by *apeglm* showing no changes, whereas a more relaxed shrinkage by *ashr* captures large fold changes, some of which are driven by single samples. To give more confidence to DEGs captured by *ashr*, more stringent filtering and post hoc validation may be warranted to select candidates for external validation.

## 5. Conclusions

Here, we present the deep sequencing of male and female DRG subtypes after SNI as a resource to the field (https://livedataoxford.shinyapps.io/drg-directory/). We show that in addition to stereotyped changes after injury, neuronal populations undergo subpopulation-specific changes at a molecular level and these vary with time. In naïve states, we see subpopulation-specific sexual dimorphism that is retained in injured states. Taken together, these data provide a starting point for future experimentation surrounding subpopulation differences as well as stereotyped changes across time points and highlights the importance of factoring sex into these studies.

## Conflict of interest statement

The authors declare no conflicts of interest relevant to this article. More broadly, D. L. Bennett has acted as a consultant on behalf of Oxford Innovation for Amgen, Biointervene, Bristows, LatigoBio, GSK, Ionis, Lilly, Olipass, Orion, Regeneron, and Theranexus over the past 2 years. He has received research funding from Lilly and AstraZeneca. He has received an industrial partnership grant from the BBSRC and AstraZeneca.

## Appendix A. Supplemental digital content

Supplemental digital content associated with this article can be found online at:http://links.lww.com/PAIN/B823http://links.lww.com/PAIN/B824http://links.lww.com/PAIN/B825http://links.lww.com/PAIN/B826http://links.lww.com/PAIN/B827http://links.lww.com/PAIN/B828http://links.lww.com/PAIN/B829http://links.lww.com/PAIN/B830http://links.lww.com/PAIN/B831http://links.lww.com/PAIN/B832http://links.lww.com/PAIN/B833http://links.lww.com/PAIN/B834http://links.lww.com/PAIN/B835http://links.lww.com/PAIN/B836http://links.lww.com/PAIN/B837http://links.lww.com/PAIN/B838http://links.lww.com/PAIN/B839http://links.lww.com/PAIN/B840http://links.lww.com/PAIN/B841http://links.lww.com/PAIN/B842.

## Supplemental video content

A video abstract associated with this article can be found at: http://links.lww.com/PAIN/B930

## Supplementary Material

SUPPLEMENTARY MATERIAL
