# Supplementary material for: Deep RNA-seq of male and female murine sensory neuron subtypes after nerve injury
Source: Pain. 2023 Jun 6;164(10):2196–215. doi: 10.1097/j.pain.0000000000002934 (PMC10502896; doi:10.1097/j.pain.0000000000002934)

Supplemental Figure 1

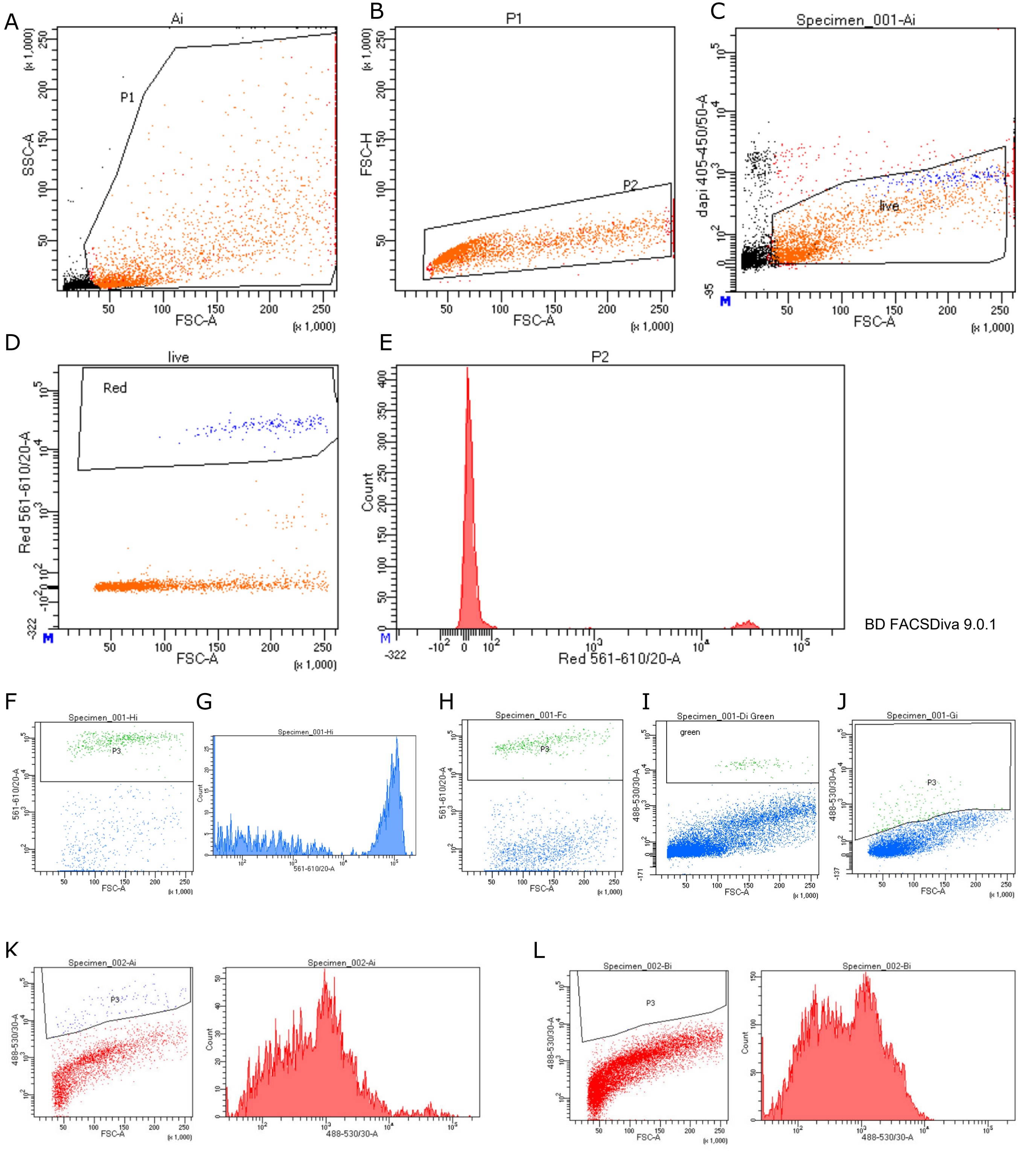

Supplemental Figure 2

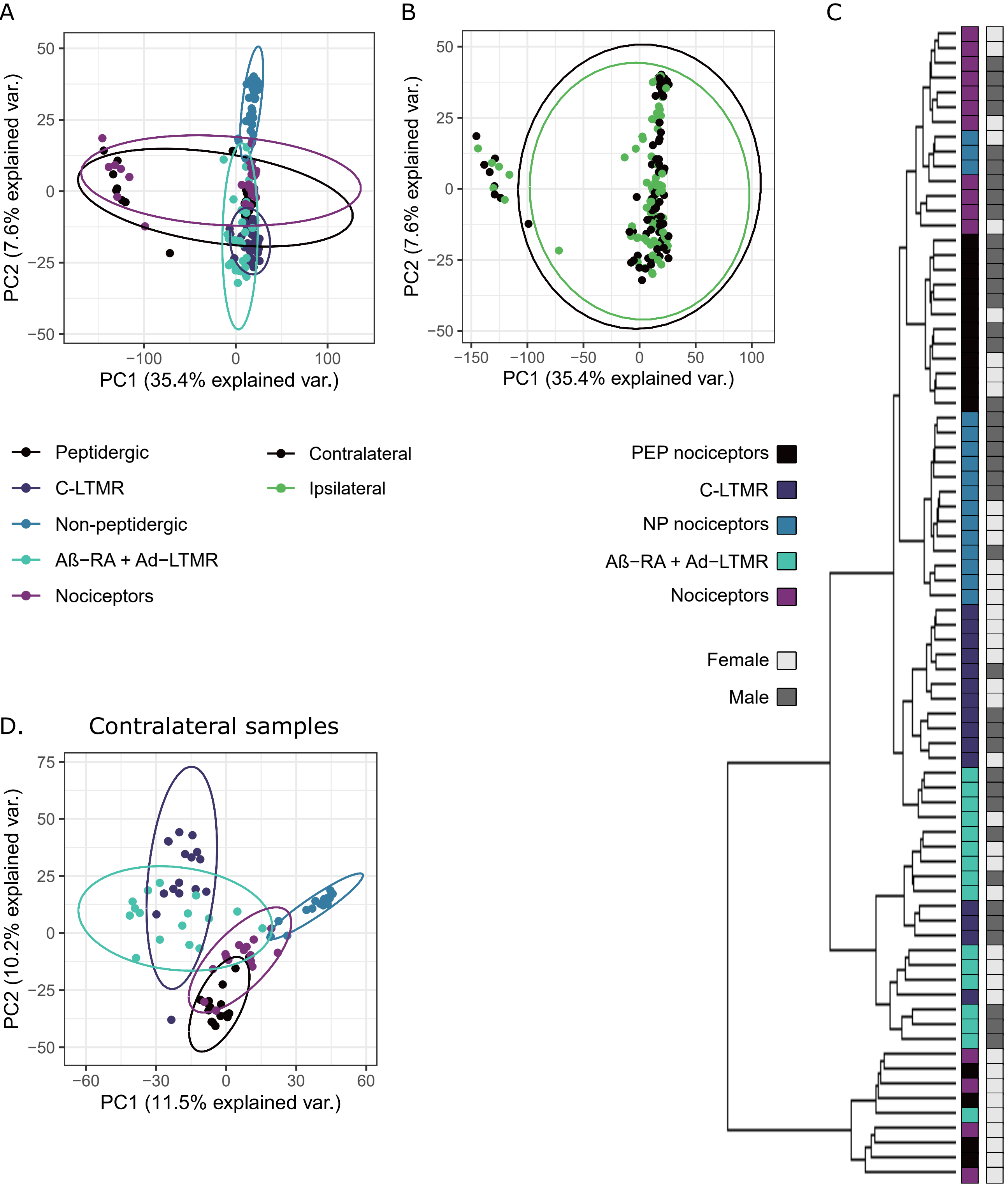

Supplemental Figure 3

A Nav1.8 enrichment or depletion (DTA/control) in male mice

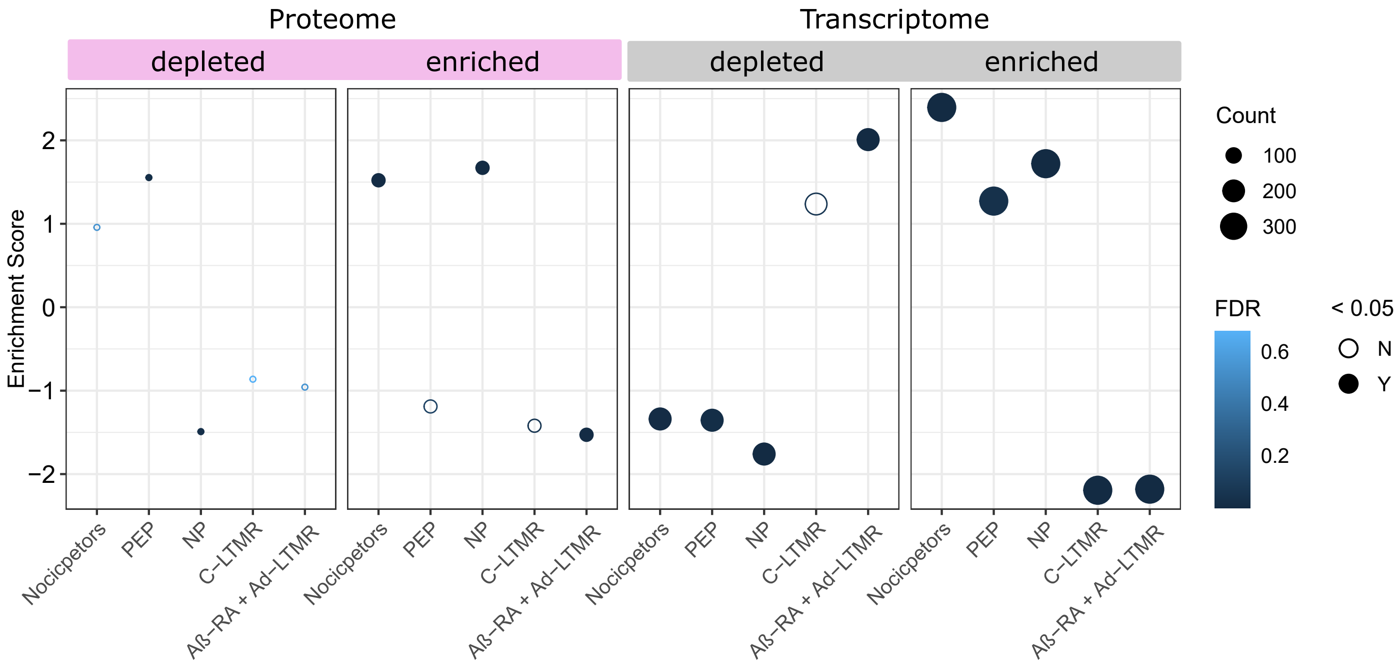

B

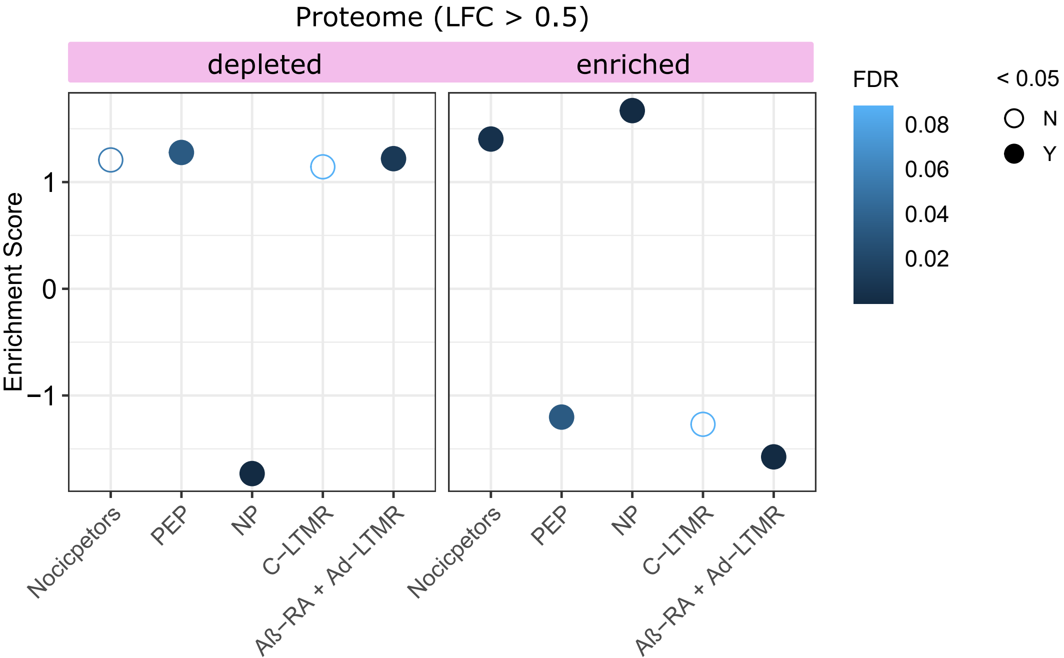

Supplement: SUPPLEMENTARY MATERIAL [file jop-164-2196-s001.pdf]
